# Spermidine Enhanced Free Polyamine Levels and Expression of Polyamine Biosynthesis Enzyme Gene in Rice Spikelets under Heat Tolerance before Heading

**DOI:** 10.1038/s41598-020-64978-2

**Published:** 2020-06-02

**Authors:** Rong Zhou, Qijuan Hu, Qiong Pu, Moxian Chen, Xiuru Zhu, Cong Gao, Guixiang Zhou, Lijun Liu, Zhiqing Wang, Jianchang Yang, Jianhua Zhang, Yunying Cao

**Affiliations:** 1 College of Life Sciences, Nantong University, Nantong, Jiangsu China; 2 Shandong Agriculture and Engineering University, Jinan, Shandong China; 3 Shenzhen Institute of Synthetic Biology, Shenzhen Institutes of Advanced Technology, Chinese Academy of Sciences, Shenzhen, China; 4 State Key Laboratory of Crop Biology, College of Life Science, Shandong Agricultural University, Taian, Shandong China; 5 Southern Regional Collaborative Innovation Center for Grain and Oil Crops in China, College of Agriculture, Hunan Agricultural University, Changsha, China; 6 Win-All Hi-Tech Seed Co., Ltd, Hefei, Anhui China; 7 Key Laboratory of Crop Genetics and Physiology of Jiangsu Province, Yangzhou University, Yangzhou, Jiangsu China; 8 Department of Biology, Hong Kong Baptist University, and State Key Laboratory of Agrobiotechnology, The Chinese University of Hong Kong, Shatin, Hong Kong

**Keywords:** Plant physiology, Plant stress responses

## Abstract

High temperatures (HT) before heading strongly inhibit the development of spikelets in rice. Spermidine (Spd) can improve rice’s resistance to HT stress; however, the mechanism underlying this effect has not been elucidated. This study investigated several parameters, including yield, superoxide anion (O_2_^.-^), protective enzyme activities, and polyamine content, in a heat-sensitive genotype, Shuanggui 1. The yield and yield components decreased dramatically when subjected to HT stress, while this reduction could be partially recovered by exogenous Spd. Spd also slowed the generation rate of O_2_^.-^ and increased protective enzyme, superoxide dismutase (SOD) and catalase (CAT) activities both under normal and high temperatures, which suggested that Spd may participate in the antioxidant system. Furthermore, genes involved in polyamine synthesis were analyzed. The results show that HT before heading significantly increased the expression of arginine decarboxylase *OsADC1*, Spd synthase *OsSPDS1* and *OsSPDS3* and had little effect on the expression of the S-adenosylmethionine decarboxylase *OsSAMDC2* and ornithine decarboxylase *OsODC1*. In addition, exogenous Spd considerably reduced the expression of *OsSAMDC2*, *OsSPDS1* and *OsSPDS3* under HT but not the expression of *OsADC1*. The above mentioned results indicate that the exogenous Spd could help young rice spikelets to resist HT stress by reducing the expression of *OsSAMDC2*, *OsSPDS1* and *OsSPDS3*, resulting in higher levels of endogenous Spd and Spm, which were also positively correlated with yield. In conclusion, the adverse effect of HT stress on young spikelets seems to be alleviated by increasing the amounts of Spd and Spm, which provides guidance for adaptation to heat stress during rice production.

## Introduction

High temperature is considered to be one of the primary environmental stress factors that affect crop growth and development. Surface air temperature is predicted to be approximately 2∼4.5 °C higher at the end of the century^1^. In addition, extreme temperature events, including high-temperature (HT) stress, will occur more frequently worldwide^2^. When subjected to HT over normal temperature (NT), the life processes of crop will be severely affected, leading to reduced yields^3,4^.

Rice (*Oryza sativa* L.) is one of the most important staple food crops and feeds approximately 65% of the population in China^5,6^. HT stress hinders its growth at any time during the crop life cycle but particularly during reproductive development^7,8^. The meiosis stage is one of the most sensitive periods to stress in rice^9^, including HT stress^10^. Before heading, including meiosis, HT stress causes abnormalities in both male and female organs, pollen abortion, low pollen germination rate on stigma and impaired pollen tube growth in self-pollinated cereals, leading to spikelet sterility, a reduction in seed-setting and a low yield^11–13^. However, the underlying biochemical mechanism by which abnormal flower organs leads to low yield has not been determined.

Polyamines (PAs) are aliphatic cations that exist in almost all living organisms as important modulators related to plant development, differentiation, stress resistance, senescence and germination^14–16^. Major polyamine species are diamine putrescine (Put), triamine spermidine (Spd) and tetramine spermine (Spm) in plants, and several key enzymes are involved in their biosynthesis. For example, biosynthesis of Put, either directly from ornithine or indirectly from arginine via agmatine, is catalyzed by ornithine decarboxylase (ODC) and arginine decarboxylase (ADC); aminopropyl groups are indicated by decarboxylated S-adenosylmethionine (SAM), which is produced from SAM by S-adenosylmethionine decarboxylase (SAMDC); the addition of aminopropyl groups transfer Put into Spd and Spm, which is catalyzed via Spd synthase (SPDS) and Spm synthase (SPMS), respectively. Activity and/or the transcriptional level of some key enzymes involved in polyamine biosynthesis could be induced by abiotic stress. For instance, an increase in ADC activity was observed in rice seedlings under salinity^17,18^, as well as in Arabidopsis under low temperature and dehydration^19^. At the transcriptional level, SAMDC expression was induced by different abiotic stresses^20^. Free polyamine and gene expression levels of encoding polyamine biosynthesis enzymes were measured in rice seedling leaves under salt stress^21^. However, the transcript levels of ADC, ODC, SPMS and SAMDC in spikelets under heat stress before heading were not determined in rice.

Some researchers hypothesized that the PAs content (including conjugated and free polyamine levels) was highly different between normal and aborting kernels^22^. Further study in divergent plant species has shown that PAs play predominant roles in responding to environmental stress^15,23^. In addition, the application of exogenous PAs can improve resistance to diverse stresses in plants. In tomato seedlings, Spd triggers effective protection under salinity-alkalinity stress, probably by maintaining the structural integrity of chloroplasts and alleviating oxidative damage^24^. It has been suggested that exogenous Spd may activate the antioxidant defense system and proline metabolism to protect white clover from water stress^25^. PAs were also reported to enhance the tolerance to heat stress^26^. The transgenic *SAMDC* tomato accumulated 1.7- to 2.4-fold higher levels of Spd and Spm than wild-type plants and presented significantly higher tolerance to HT stress compared to wild-type plants^27^. Compared with the stress-sensitive genotype, stress-tolerant rice generally has a large capacity to enhance the biosynthesis of polyamine in response to heat stress^28^. To date, the physiological role of PAs in tolerance to environmental stress has not been determined^29^. However, there is little information about the relationship between internal polyamine accumulation and spikelet sterility before heading in rice under heat stress.

In this study, we investigated the polyamine content and expression levels of genes encoding enzymes involved in polyamine biosynthesis in a heat-sensitive genotype, Shuanggui 1, under HT stress before heading and explored the possible correlations between spikelet sterility, polyamine content, gene expression level and heat sensitivity of the two cultivars by application of exogenous substances.

## Materials and methods

### Plant materials and growth conditions

The experiment was undertaken in Life and Science of College, Nantong University, Jiangsu Province, China (32^0^ 1´ N, 120^0^ 53´ E). One *indica* rice (*Oryza sati*va L.) genotype, SG-1 (Shuanggui 1) was cultivated. Thirty-day-old seedlings in the paddy land outside on 5 May were then transplanted to plastic pots (40 cm in height and 29 cm in diameter with 22-kg sieved soils). There were 3 hills in each pot with 1 seedling per hill. Fertilizers of urea (2.5 g) and KH_2_PO_4_ (0.5 g) were applied to each pot before transplanting, and another 1.0 g and 0.5 g urea were employed as fertilizer at mid-tillering stage and panicle initiation stage, respectively. The water layer was kept at a height of 1–2 cm during the entire growing stage. SG-1 headed on 5 August (50% of plants), flowered on 7 August and was harvested on 3–4 October.

### Spd and heat stress treatment

**Figure 1:**
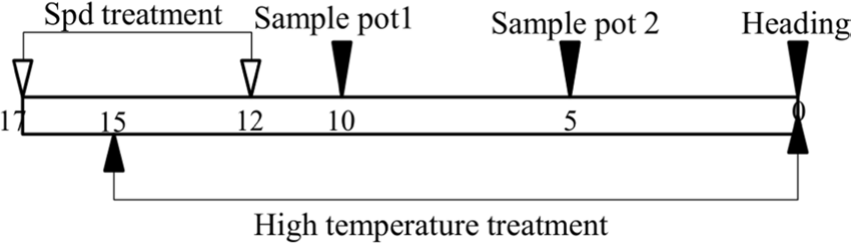
The method of Spd and high temperature treatment, the sample pots.

The experiment was processed with a 2-by-2 factorial design with four treatments (two temperature treatments: NT-normal temperature and HT-high temperature; two levels of spd treatments: injected and not injected). Twenty pots were set as replicates in each treatment and completely randomly arranged. From 15 days before heading to preliminary heading stage (Fig. 1), plants were subjected to HT treatments according to Cao *et al*.^30^ with minor modifications.

Starting on the second day before HT treatment (Fig. 1), 1 × 10^−3^ mol L^−1^ Spd was applied to the panicles of the main stems by 1-ml syringe and injected carefully from the top into the boot of the flag leaf. Spd (0.5 ml) was applied to each panicle for 5 days. The Spd solution contained ethanol and Tween-20 at final concentrations of 0.1% (v/v) and 0.01% (v/v), respectively. Meanwhile, the same volume of deionized water with the same concentration of ethanol and Tween-20 was applied to the control plants.

As for HT treatment, half the amount of pots with injected and no-injected Spd were moved and grown in the phytotron (5 m × 5 m) from the onset of pollen mother cell (PMC) meiosis (the distance between the sheath of the flag leaf and the penultimate leaf was 10–11 cm, 15 days before heading) to preliminary heading stage according to Ding^31^.

The temperature treatment was performed with an automatic control system (Zhejiang Qiusi). The temperature of HT was set in a cycle as 29.0 °C from 6:00 to 8:00, 34.0 °C from 8:00 to 10:00, 39.0 °C from 10:00 to 14:00, 34.0 °C from 14:00 to 18:00 and 29.0 °C from 18:00 to 6:00 of the next day. The temperature of the control was 27.0 °C from 6:00 to 8:00, 30.0 °C from 8:00 to 10:00, 33.0 °C from 10:00 to 14:00, 30.0 °C from 14:00 to 18:00 and 27.0 °C from 18:00 to 6:00 the next day. Other parameters were set to be identical between the NT and HT treatment, such as relative humidity (RH) held at 45% ± 5% constantly^32^; the concentration of CO_2_ was maintained at 380 ± 15 μmol mol^−1^; and light intensity was 300–600 μmol m^−2^ s^−1^ from 6:00 to 8:00, 600–900 μmol m^−2^ s^−1^ from 8:00 to 10:00, 900–1200 μmol m^−2^ s^−1^ from 10:00 to 14:00, 900–600 μmol m^−2^ s^−1^ from 14:00 to 16:00, and 600–300 μmol m^−2^ s^−1^ from 16:00 to 18:00.

## Sampling

At the tillering stage, one hundred main stems in each treatment were tagged; among these stems, twenty panicles at 5 days (Fig. 1) after HT treatment were picked for measurement of gene expression. Another eighty panicles were sampled at 5 and 10 days (Fig. 1) after HT treatment for analysis of free polyamine (Put, Spd, and Spm) levels. The flag leave was sampled at 5 and 10 days after HT treatment to measure the production rate of O_2_^−^ and protective enzyme activities, including peroxidase (POD), superoxide dismutase (SOD), and catalase (CAT). Samples for analysis of gene expression were frozen in liquid nitrogen and stored at −80 °C.

### Measurement of grain yield and yield components

At the maturity stage, five pots of each treatment were applied for calculation of grain yield, and the two were analyzed for grain weight, spikelet number (per panicle), percentages of sterile spikelets, partially filled grains and fully filled grains.

### Determination of free polyamine levels

Free polyamines were extracted and quantified following the method of Yang^15^ with minor modifications. Fresh panicles (0.5–1.0 g) were homogenized in 5 mL 5% (v/v) cold perchloric acid and incubated for 1 h at 4 °C. 1,6-Hexanediamine was added to the homogenates as the internal standard, and the recovery percentages of Put, Spd and Spm were 99.5 ± 2.6%, 99.8 ± 1.8% and 102.5 ± 2.6%, respectively. The homogenates were centrifuged at 15,000 g for 30 min, and the supernatants were collected for analysis of free polyamines. PAs in the supernatants were subjected to benzoylation in alkaline medium. Diethyl ether was applied to extract benzoyl-PAs and then evaporated in a water bath (50–60 °C). The residue was dissolved in methanol and benzoyl polyamines separated by HPLC. Twenty microliters of the prepared extract was injected into a 50 µL loop and loaded onto a reversed-phase column (C18, 4.6 mm × 100 mm, 5 µm particle size, Inertsil. ODS-3). and then eluted through the column with 64% methanol at a flow rate of 1.0 mL min^−1^. Polyamine peaks were detected with a Shimadzu UV–vis spectrophotometric detector at 254 nm. The PA levels were determined from the standard curves developed from standard PAs (Put, Spd and Spm) and expressed as nmol g^−1^ FW. Soluble conjugated PAs were calculated by subtracting the free PAs from the acid-soluble PAs.

### Analysis of the generation rate of O_2_^−^ and protective enzyme activities

The generation rate of O_2_^−^ was measured using the hydroxylamine oxidation method according to Wang^33^ with minor modifications. Next, 0.5∼1.0 g of fresh flag leaves was weighed, cut into small pieces, ground with a mortar and pestle, and collected in 7 ml of 0.05 mol L^−1^ phosphate buffer (PBS), pH 7.0. After homogenate centrifugation (10,500 × g, 20 min at 4 °C), 1 mL of the supernatant fractions, 1 mL PBS and 2 mL hydroxylamine were kept warm at 25 °C for 1 hour, extracted with an equal volume of ether, and centrifuged at 1500 × g for 5 min; next, 1 mL water phase, 1 mL of 7 mmol L^−1^ sulfanilic acid and 1 mL of 7 mmol L^−1^ α-naphthylamine were kept warm at 25 °C for 20 min to measure the absorbance at λ = 530 nm. As a control, the experiment was was repeated with PBS instead of a sample.

To prepare crude enzyme extracts, 500 mg of fresh flag leaves were weighed, cut into small pieces, ground with a cooled mortar and a pestle, and collected in 5 mL of 0.1 mmol L^-1^ phosphate buffer, pH 7.5. After homogenate centrifugation (12,000 × g, 20 min at 4 °C), the supernatant fractions were used for the determination of POD, SOD and CAT activities. The activity of POD was measured by guaiacol oxidation in a 4-mL reaction system including 1.95 mL 0.2% H_2_O_2_, 0.95 mL 0.2% guaiacol, 1 mL PBS (pH 7.0, 50 mmol L^−1^) and 1 mL crude enzyme at λ = 470 nm. An increase of absorbance by 0.01 per minute was defined as 1 unit (U). The activity of SOD was measured by the nitroblue tetrazolium (NBT) at λ = 560 nm according to Grigore^34^. The amount of enzymes inhibiting 50% photoreduction of NBT is one unit of enzyme activity (U). The activity of CAT was measured by the ultraviolet absorption method in a 3-mL reaction system including 1 mL 0.2% H_2_O_2_, 1.9 mL H_2_O and 0.1 mL crude enzyme at λ = 240 nm. A decrease in absorbance by 0.01 per minute was defined as 1 unit (U).

### Gene expression

The expression levels of genes involved in polyamine synthesis were measured by Quantity Real Time PCR (qRT-PCR). The primers used for qRT-PCR were designed with Prime3 Plus software, as shown in Table 1, and *OsACTIN* was used as an internal control. According to the manufacturer’s instructions, total RNA was extracted with TRIzol (Invitrogen) and synthesized to first-strand cDNA using the PrimeScript RT reagent kit with gDNA Eraser (Takara). The PCR amplifications were performed with the SYBR green method on the Roche Real-time PCR system^35^. The qRT-PCR program was 30 s at 95 °C followed by 40 cycles of 5 s at 95 °C and 34 s at 60 °C. According to the 2^−∆∆t^ method^36^, expression levels were determined by the cycle threshold (C) value of each sample. All experiments were repeated at least four times.

**Table 1:** RT-qPCR Primers used in the study.

Gene name	Primer	Sequence(5′-3′)	Amplicon size (bp)
*OsADC1*	F	CACCTCCTTCTCCTCCTCCA	120
	R	ACGACCACCATGACACGATACAACC	
*OsODC1*	F	GACGAGGTGGTGAGGGGTTA	152
	R	TAGCAGGAGTAGGCGAGGTAGG	
*OsSAMDC2*	F	TCGGCTACAGCATTGAGGAC	234
	R	GCCAGAGATGAGGAAGAAAGGAA	
*OsSPD1*	F	ACTGAGCCCAAGGCCAACT	190
	R	GTAAGGGAGATGCGGGAAAAC	
*OsSPD3*	F	AGGGGAAGTCACCATACCAAGA	188
	R	ACCATCACCACCTCCAATAACC	
*OsACTIN*	F	CTTCATAGGAATGGAAGCTGCGGGTA	197
	R	CGACCA CCTTGATCTTCATGCTGCTA	

### Data analysis

Statistical analyses of all data were performed using analysis of variance (ANOVA) with the SAS/STAT system (SAS Institute, USA). Values from different parameters were used to calculate the means and separated using Duncan’s test (α = 0.05). Different parameters were also used to calculate the standard deviation and are presented as error bars in the graphs. In addition, correlation analysis was used to evaluate the relationships between PAs in the young spikelets and the number of differentiated spikelets per panicle, the percentage of degeneration or sterility, 1000-grain weight.

## Results

### Spd improved the grain yield and yield components under HT treatment

As shown in Table 2, Spd significantly increased the yield of SG-1 in both the NT and HT treatments compared with the control. Spd mainly significantly increased grain number per spike (4.9% in NT, 12.2% in HT), grain weight (1.5% in NT, 1.1% in HT) and seed-setting rate, including partially filled grains (16.2% in NT, 33.3% in HT) and fully filled grains (3.4% in NT, 9.5% in HT), and reduced sterile spikelets per panicle (29.4% in NT, 36.8% in HT). Compared with NT, Spd treatment presented more improvement in yield under HT stress. This result may be observed due to the production limit at NT, where there is little space for improvement; while the yield is considerably lower under the HT stress, and there still had buoyant space. Therefore, it is indicated that the application of Spd can mitigate the injury to HT stress before heading.

**Table 2:** Effect of Spd application on grain yield and yield components at different temperatures before heading.

Temperature	Spd	Grain yield	Spikelet number (panicle^−1^)	Sterile spikelets (%)	Partially filled grains (%)	Filled grains (%)	1000-grain weight (g)
NT	−Spd	86.8 ± 8.4 b	197.4 ± 4.8 b	14.3 ± 1.2 b	3.7 ± 0.4 a	82.0 ± 0.1 b	20.2 ± 0.1 b
	+Spd	96.5 ± 5.9 a	207.1 ± 5.6 a	10.1 ± 1.1 c	4.3 ± 0.2 a	84.8 ± 0.1 a	20.5 ± 0.2 a
HT	−Spd	61.4 ± 5.6 d	159.4 ± 7.8 d	22.0 ± 1.9 a	3.0 ± 0.4 a	75.0 ± 2.6 c	18.6 ± 0.2 d
	+Spd	75.1 ± 3.1 c	178.8 ± 5.2 c	13.9 ± 1.5 b	4.0 ± 0.2 a	82.1 ± 4.8 b	18.8 ± 0.1 c

Means±SDs were calculated from the data of five pots. Values followed by different letters within the same column are significantly different at P < 0.05 between the different treatments, including temperature and Spd.

### Spd decreased O_2_^−^ production rate and protective enzyme activities under HT treatment

**Figure 2:**
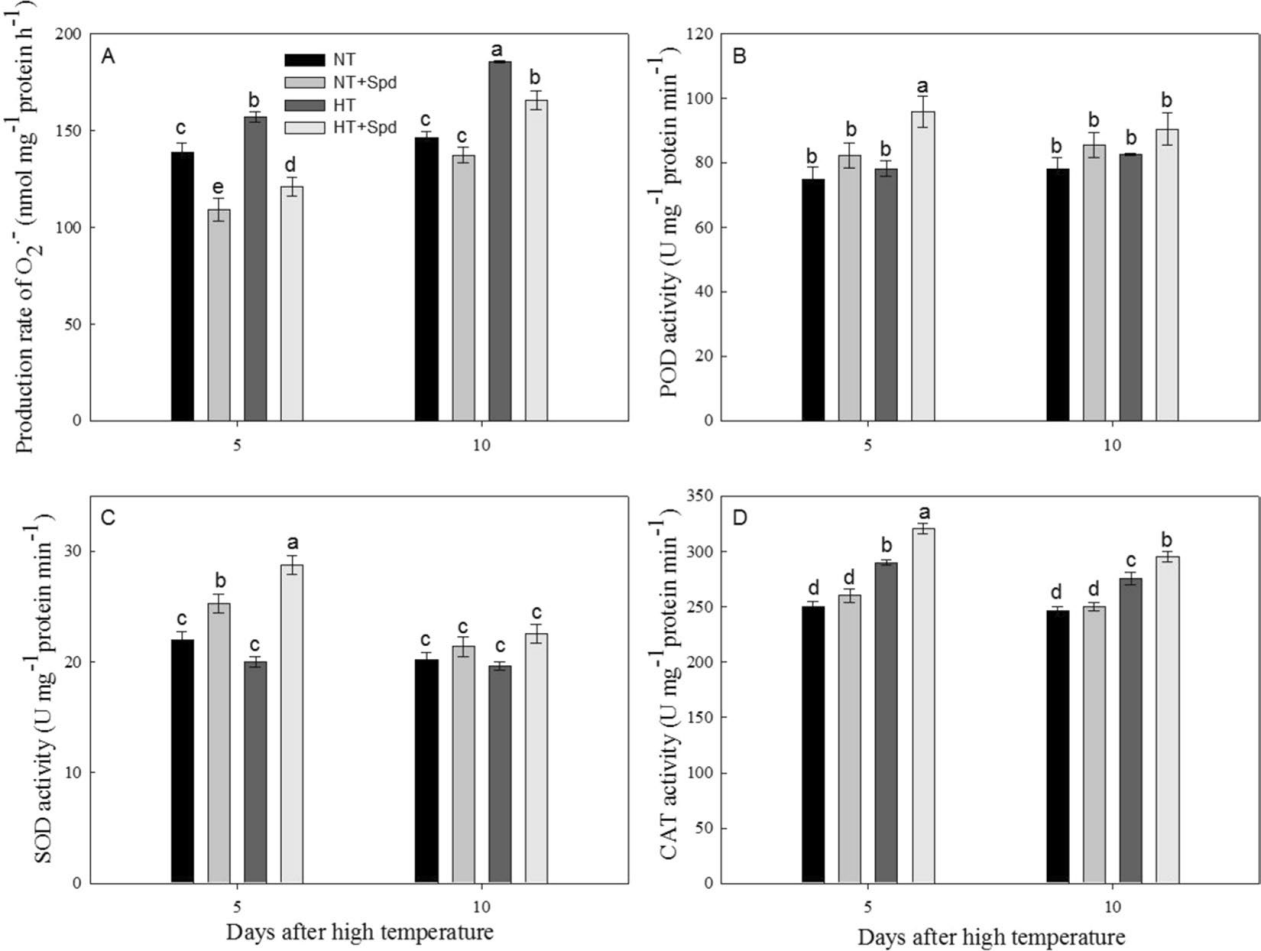
Changes in the O_2_^−^ production rate of the leaves and protective enzyme activities with or without Spd in rice upon heat stress before heading. The genotype SG-1 was pot-grown. Normal temperature (NT) and high temperature (HT) were conducted from 15 days before heading to heading. The chemicals were applied to panicles daily and lasted for 5 days starting on the 2rd day before HT stress treatment. Control plants were applied with deionized water. The data were the average of the three repeats. Different letters indicate statistical significance at *P* < 0.05 among the different treatments, including 5 and 10 days after heat stress.

The generation rate of O_2_^−^ was significantly increased by HT stress (Fig. 2A). Spd showed different levels of antioxidation under different temperature conditions. Regarding NT treatment, Spd decreased the generation rate of O_2_^−^ significantly in 5d (21.4%) and 10 d (6.0%) compared with the control, while generation of O_2_^−^ was dramatically slower both in 5d (23.1%) and 10 d (10.8%) in Spd treatment than in the control under HT stress. This result suggested that Spd could reduce the generation of O_2_^−^ under HT stress. To explain the decrease in the generation of O_2_^−^ with Spd, we analyzed the protective enzyme activities related to antioxidation (Fig. 2B–D). We found that Spd increased POD and SOD activities in 5d and CAT activities both 5d and 10d under HT stress, which concluded that the increase of protective enzyme could be the reason for the decrease of generation of O_2_^−^ with Spd.

### Effects of Spd on endogenous polyamine content and gene expression involved in polyamine synthesis

As shown in Table 3, HT stress increased the content of Spd by approximately 36.5% in 10 d and increased the content of Spm by approximately 77.4–1004.5% at both 5 and 10 d after HT treatment compared with the NT treatment and decreased the content of Put by approximately 31.2–60.1% at both 5 and 10 d after HT treatment compared with the NT treatment in young panicles. Spd increased the content of Spd and Spm by approximately 20.4–398.1% in young panicles but decreased the content of Put under HT stress. Similar results were found in NT treatment, while only Spd content on 5 d was not enhanced by Spd treatment.

**Table 3:** Effect of spermidine application on endogenous polyamines content of the young panicles at different temperatures before heading.

Temperature	Spd	Put (nmol g^-1^FW)		Spd (nmol g^−1^FW)		Spm (nmol g^−1^FW)	
		5 d	10 d	5 d	10 d	5 d	10 d
**NT**	−Spd	123.3 ± 1.6 a	32.3 ± 0.1 a	564.3 ± 3.9 a	153.2 ± 0.4 d	84.5 ± 3.2 d	54.9 ± 0.2 d
	+Spd	63.9 ± 0.1 c	19.0 ± 0.1 b	463.3 ± 2.2 b	172.9 ± 0.3 c	130.0 ± 0.7 c	79.9 ± 1.5 c
**HT**	−Spd	84.8 ± 1.2 b	12.9 ± 4.8 b	378.5 ± 1.0 c	209.1 ± 21.0 b	172.8 ± 6.7 b	97.4 ± 8.5 b
	+Spd	21.9 ± 0.5 d	8.0 ± 0.1 c	525.2 ± 4.6 a	251.8 ± 0.5 a	860.8 ± 13.0 a	153.4 ± 1.8 a

Means±SDs were calculated from the data of three repeats. Values followed by different letters within the same column are significantly different at P < 0.05 between the different treatments, including temperature and Spd. 5 d and 10 d in the table were 5 days and 10 days after HT treatment, respectively.

**Figure 3:**
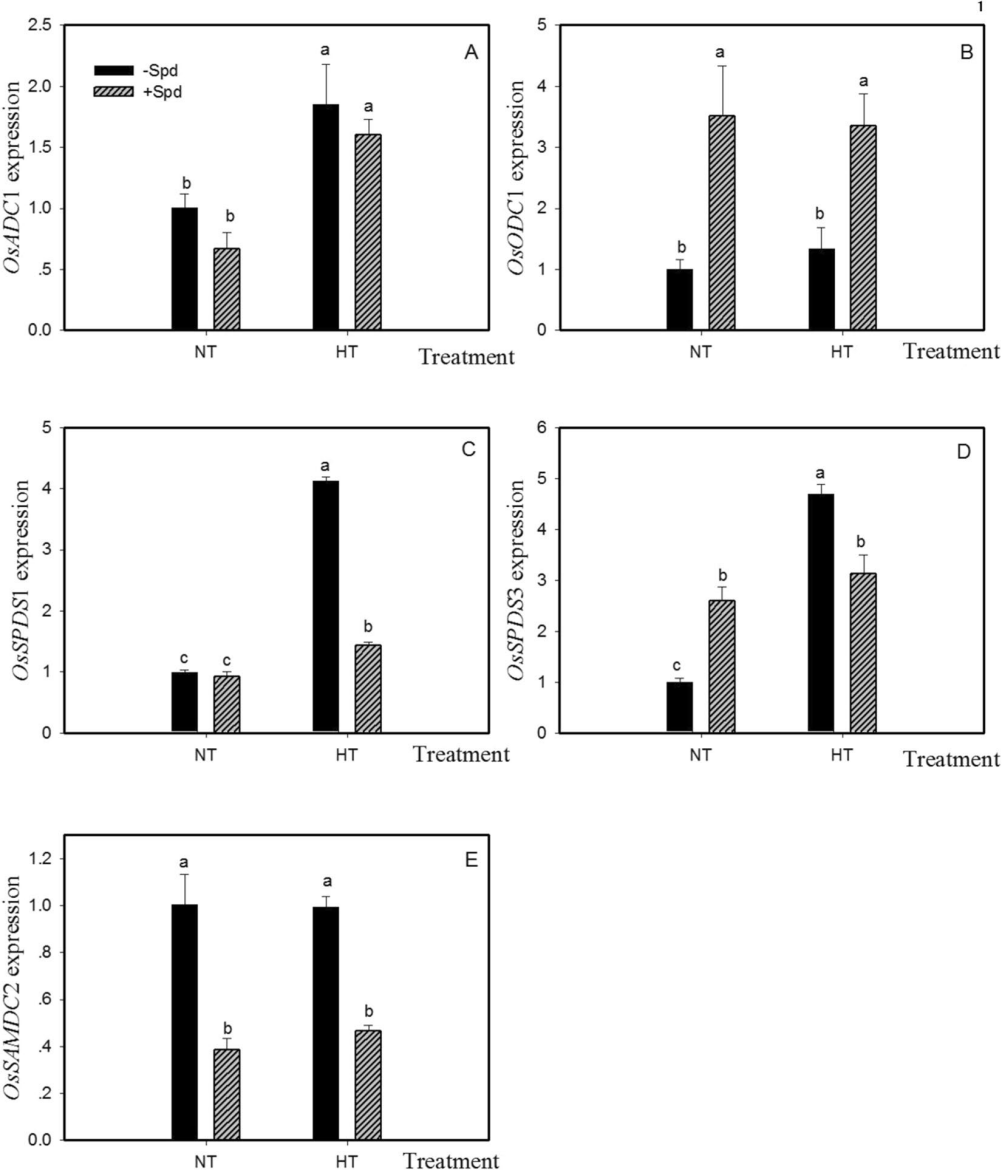
Changes in the gene expression involved in polyamine synthesis with or without Spd in rice upon heat stress before heading. The genotype SG-1 was pot-grown. Normal temperature (NT) and high temperature (HT) were conducted during meiosis. The chemicals were applied to panicles daily and lasted for 5 days starting on the 2rd day before HT stress treatment. Control plants were applied with deionized water. Gene expression analysis in panicles was determined 5 days after heat stress and had four repeats. Different letters indicate statistical significance at *P* < 0.05 between the different treatments, including temperature and Spd.

HT stress had little effect on the expression of *OsSAMDC2* and *OsODC1* but significantly increased those of *OsADC1*, *OsSPDS1* and *OsSPDS3* (Fig. 3A–E). The transcription levels of most genes changed compared with the control, except for *OsADC1*, both in NT and HT, and *OsSPDS1* in NT due to Spd application. Exogenous Spd remarkably reduced the expression level of *OsSPDS1, OsSPDS3* and *OsSAMDC* under HT treatment and increased the expression level of *OsODC1*. The above results indicated that Spd could contribute to resistance to HT stress in young rice spikelets by reducing the expression levels *OsSAMDC2*, *OsSPDS1* and *OsSPDS3*.

As the results of the correlation analysis showed (Table 4), there was a significantly positive correlation between the content of Spd and Spm and seed-setting rate and thousand grain weight (Table 4, r = 0.851**∼0.942**). The content of Put was significantly negatively correlated with the seed setting rate (r = −0.863**). The accumulation of Spd and Spm and reduced expression levels of *OsSAMDC2*, *OsSPDS1* and *OsSPDS3* were promoted by Spd, while synthesis of Put was decreased and expression levels of *OsODC1* were increased by Spd in young panicles, which helped to reduce the damage from HT stress (Table 3 and Fig. 3). Furthermore, Spd increased the seed-setting rate and the thousand grain weight, resulting in higher yield than the control under HT stress.

**Table 4:** Correlations of polyamines in young spikelets and yield components.

Correlation (r)	Spikelets number	Sterile spikelets	Fully filled grains	1000-grain weight
Put	−0.471	0.921^**^	−0.863^**^	−0.3
Spd	0.357	−0.931^**^	0.942^**^	0.891^**^
Spm	0.416	−0.914^**^	0.924^**^	0.851^**^

The two asterisks followed number in table are significantly different at P < 0.01 between the items of row and column. Resource data is from Table 2 (Spikelets number, Sterile spikelets, Fully filled grains, 1000-grain weight) and Table 3 (Put, Spd, Spm). All the data points from four treatments have three replicates.

## Discussion

Simultaneous HT stress during sensitive developmental stages increased retrograded spikelets^37^, where the percentage of sterile spikelets (retrograded spikelets) was dramatically increased and finally led to severe loss of yield^7^; a similar phenomenon was found in the present study (Table 2). Polyamines are aliphatic polycations found in almost living organisms, and it is widely reported that polyamines are essential factors for development and survival, including resistance to heat stress^28,38^. According to our results, Spd application could not only significantly increase the yield under normal temperature but could also reduce the yield loss under HT stress before heading. Therefore, Spd was confirmed to play a predominant role in yield improvement and grain sterility reduction during seed development in rice. In addition, we concluded that exogenous Spd increased endogeneous Spd and Spm level, and then increased grain weight under HT in comparison to standard HT treatment (Tables 2 and 3). Previous studies have indicated that although higher temperature increases grain filling rate, it will dramatically shorten grain filling period. Thus, the overall grain weight is reduced under high temperature^5^. In this study, Spd content was firstly reduced and then elevated, whereas Spm content was consistently increased over time during HT treatment, showing similar changes of polyamines content as existing reports related to water stress^38^. Therefore, the grain weight increment under exogeneous Spd treatment may further elevate internal polyamine contents, thus resulting in higher grain weight during HT stress.

PAs have been frequently described as endogenous plant growth regulators or intracellular messengers, which mediate many physiological processes, including abiotic stress responses^38,39^. In addition, plants respond to stress with fast and sensitive changes in reactive oxygen species (ROS) contents, which play key roles in metabolomics and signal transduction^40^. Excessive accumulation of ROS can lead to severe damage to the cellular plasma membrane and organelle, eventually interfering with plant growth and development^25,41^. Spd was reported to play a key role in stress defense and ROS scavenging. For instance, a lower generation rate of O_2_^−^ and enhanced antioxidant enzyme activities were observed in Spd-treated white clover seeds under water stress, which further confirmed the role of Spd as ROS scavengers^25^. Similar results were found in the present study, demonstrating HT stress clearly stimulated the generation of O_2_^−^ and the increase in CAT activity, and application of exogenous Spd strongly reduced the production of O_2_^−^ and enhanced POD, SOD and CAT activities (Fig. 2). For instance, under osmotic stress, exogenous PAs inhibited the accumulation of O_2_^−^ and H_2_O_2_ in wheat seedlings and barley leaves^42^. It is suggested that Spd could reduce the accumulation of O_2_^−^ and improve the resilience of rice plants, ultimately remitting the damage from heat stress. In addition, Spd led to different production rates of O_2_^−^ under normal temperatures, where Spd slowed the generation of O_2_^−^ on the 5th day after normal temperature treatment, but this effect did not extend to the 10th day, which may relate to the action of the autogenous self-balance structure of ROS and resulted in a relatively normal generation rate of O_2_^−^ under ordinary circumstances.

In plants, ADC, ODC, SAMDC and SPDS are key biosynthetic enzymes that play key roles in the synthesis of polyamines. Among them, the activities of ADC and ODC affect the generation of Put directly, SAMDC is a rate-limiting enzyme in the synthesis of Spd and Spm, and SPDS affects the synthesis of Spd directly. Generally, these polyamine synthetases respond to stress, eventually leading to changes in polyamine content.

As previous studies showed, after low-temperature treatment, the expression levels of *OsADC*, *OsSAMDC* and *OsSPDS* in potato leaves were upregulated, while the change in polyamine content was not significant^43^. Under long-term salt stress, the activity and transcription level of ADC increased in salt-tolerant rice cultivars and decreased in salt-sensitive cultivars^17^. As shown in the present study, HT increased not only the expression levels of *OsADC1* but also *OsSPDS1* and *OsSPDS3* in young spikelets of heat-sensitive cultivars; however, HT exerted little influence on *OsODC1* and *OsSAMDC2*, ultimately increasing the levels of endogenous Spd and Spm.

Exogenous PAs were thought to contribute to changes in the activities and gene expression levels of polyamine synthetases in plants, thereby resisting the adverse environment. The increase of SAMDC activity was suppressed by HT stress, which led to poor performance of tomato pollen, but this reduction could be partially restored by using exogenous Spd^44^. These results indicated that Spd was closely related to the heat tolerance of tomato. In this study, the expression levels of *OsSAMDC2*, *OsSPDS1* and *OsSPDS3* decreased after the application of exogenous Spd under HT stress, finally increasing the internal contents of Spd and Spm to a significantly higher level than that observed without Spd application. In addition, similar changes in PAs content were observed under NT treatment, the content of Put decreased in both the NT and HT treatments.

According to the results of correlation analysis between PAs contents and yield components, various PAs may play different roles in HT tolerance. For instance, Put appears to be inversely correlated with yield components, including sterile spikelets, percentages of fully filled grains and thousand grain weight, and Put was also decreased by exogenous Spd both under NT and HT. In contrast, Spd and Spm were positively correlated with the above mentioned two yield components, as well as grain weight.

In conclusion, it is suggested that the adverse effect of HT stress on young spikes is alleviated by increasing the amount of Spd and Spm, while the resistance mechanisms of Spd on HT stress warrants further study. The present results also provide a fast and efficient means of alleviating the damage caused by HT stress during the meiosis stage of young spikes of rice, which may contribute to the regulation of rice cultivation. Furthermore, corporation of innovative multi-omics technology may help us unravel the molecular dynamics of this interesting research topic^45,46^.
